# Bacterial type IV secretion system induces specific and nonspecific protective immunity

**DOI:** 10.1128/mbio.00448-25

**Published:** 2025-06-25

**Authors:** Fernanda V. S. Castanheira, Marcelo S. F. Pereira, Marco A. Ataide, Danielle P. A. Mascarenhas, Rhanoica O. Guerra, Gustavo F. S. Quirino, Fausto Almeida, Dario S. Zamboni

**Affiliations:** 1 Departamento de Biologia Celular e Molecular e Bioagentes Patogênicos, Faculdade de Medicina de Ribeirão Preto, Universidade de São Paulo, Ribeirão Preto, São Paulo, Brazil; 2 Departamento de Bioquímica e Imunologia, Faculdade de Medicina de Ribeirão Preto, Universidade de São Paulo, Ribeirão Preto, São Paulo, Brazil; Institut Pasteur, Paris, France

**Keywords:** *Legionella pneumophila*, type IV secretion system, infection, immunity, innate instruction of adaptive immunity

## Abstract

Pathogenic microbes trigger rapid and robust innate immune responses that effectively restrict pathogen replication and promote long-lasting adaptive immunity. The differential recognition of pathogenic versus non-pathogenic microbes occurs through the detection of host cell damage or pathogen molecules secreted via virulence factors, such as specialized bacterial secretion systems. Despite the well-established role of bacterial secretion systems in pathogenesis and subversion of host cell responses, their impact on immune activation remains largely unexplored. Here, we used *Legionella pneumophila*, an intracellular bacterial pathogen that expresses the Dot/Icm type IV secretion system (T4SS), to assess the importance of T4SS in inducing specific and nonspecific long-lasting immunity. Using thymidine auxotrophic *L. pneumophila* strains that are competent for T4SS expression but unable to multiply in mouse tissues, we found that infection with T4SS-sufficient bacteria, but not T4SS-deficient bacteria, induces protective immunity. Mechanistically, this process requires MyD88 signaling but not individual TLRs, Trif, TNF-α, IFN-γ, IL-12, IL-17, IL-23, or inflammasomes. CCR2^+^ monocytes and CD4^+^ T cells were partially involved in T4SS-induced responses that conferred protection against secondary infections with *L. pneumophila*, *Legionella longbeachae*, and the pathogenic fungus *Cryptococcus neoformans*. Our findings contribute to the identification of pathogen determinants that induce specific and nonspecific immunity, a process central to understanding host-pathogen interactions and with potential implications for vaccine development.

**IMPORTANCE:**

Understanding how bacteria interact with the immune system is crucial for developing better treatments and vaccines. This study reveals that a bacterial secretion system, the type IV secretion system (T4SS) of *Legionella pneumophila*, triggers a targeted immune response but also enhances broader, nonspecific immunity. Using advanced infection models, the research shows that T4SS-driven immunity protects against multiple pathogens, including bacteria and fungi, even in the absence of traditional immune signaling pathways. These findings suggest that bacterial secretion systems can serve as novel tools for training the immune system, with potential applications in vaccine development and immunotherapy. By uncovering new ways bacteria influence immune memory, this work advances our understanding of host defense mechanisms and opens new avenues for designing strategies to enhance protection against infectious diseases.

## INTRODUCTION

*Legionella pneumophila* is a Gram-negative, facultative intracellular bacterium that thrives in freshwater environments and, once inhaled by humans, can cause Legionnaires’ disease (1, 2). Upon human infection, *L. pneumophila* infects and replicates within alveolar macrophages by preventing the fusion of early and late endocytic organelles with the vacuole in which it resides. This process is driven by the secretion of multiple effector proteins directly into the cytoplasm via the Dot/Icm type IV secretion system (T4SS) (3–5). The Dot/Icm system consists of approximately 26 genes that encode a diverse set of proteins, and its expression is essential for bacterial multiplication in bone marrow-derived macrophages (BMDMs) and in mice (6, 7).

The innate immune response against *L. pneumophila* is initiated by the activation of pattern recognition receptors (PRRs), such as Toll-like receptors (TLRs) and Nod-like receptors (NLRs), culminating in NF-κB and MAPK signaling and cytokine production. Among PRRs, Toll-like receptors such as TLR2, TLR4, and TLR9 play a crucial role in the early recognition of bacterial components (8–10). These receptors signal via MyD88 and TRIF to upregulate numerous genes, leading to robust neutrophil recruitment (10–12). In addition to membrane-bound TLRs, *Legionella* molecules that reach the host cytoplasm, such as flagellin and LPS, activate intracellular signaling pathways, including the NAIP5/NLRC4 inflammasome and the caspase-4/11-mediated noncanonical activation of the NLRP3 inflammasome (13–17). Once activated, the NLRC4 inflammasome triggers robust caspase-1 activation, leading to cell death, production of inflammatory cytokines such as IL-1β and IL-18, inflammation, and restriction of bacterial replication (reviewed in reference 18). The inflammatory response triggered by *L. pneumophila* initiates a strong adaptive immune response in the host. In mouse models of infection, CD4^+^ T cells differentiate into Th1/Th17 effector cells in the lung through a MyD88- and inflammasome-dependent process (19, 20). The Dot/Icm type IV secretion system is required for efficient bacterial replication and is therefore expected to play a key role in activating both innate and adaptive immune responses (20, 21; reviewed in reference 22). One of the main features distinguishing adaptive from innate immunity is the specificity of the response against a particular pathogen. Upon exposure to a given pathogen or its components, the adaptive immune system is activated and responds more rapidly upon subsequent encounters with the same agent (23). However, it has been demonstrated that innate immunity can also mount a rapid and strong response upon a second infection. In this case, the response is not necessarily specific to the same pathogen or antigen but can also be triggered by unrelated pathogens.

It is well accepted that the presence of virulence determinants, such as specialized bacterial secretion systems, triggers cytosolic sensors, enabling the recognition and discrimination of pathogenic versus non-pathogenic microbes (reviewed in reference 24). However, the role of specialized virulence factors in the activation of trained and adaptive immune responses remains largely unexplored. Given that the *Legionella pneumophila* Dot/Icm system is a key virulence factor essential for activating intracellular sensors, we used auxotrophic *Legionella* mutants, either T4SS-sufficient or T4SS-deficient, to evaluate the importance of specialized bacterial secretion systems in generating adaptive immunity in mice. Our data show that the *L. pneumophila* T4SS is essential for inducing both antigen-specific and nonspecific immunity against subsequent infections. These findings contribute to our understanding of how protective immunity is specifically generated against pathogenic organisms, with potential implications for the development of vaccines.

## MATERIALS AND METHODS

### Bacteria

*Legionella pneumophila* strains used included *Lp thyA* (Lp02) and the *dotA^-^ thyA* (Lp03), *flaA^-^ thyA* isogenic mutants. Challenge experiments were performed using *flaA^-^ L. pneumophila* in the JR32 background (25). Bacteria were grown on buffered charcoal-yeast extract (CYE) agar as described (26). The *thyA* mutant was grown on BCYE agar supplemented with 100 mg/mL thymidine (BCYET). *L. longbeachae* strain NSW150 was also used in this study (27). Before infection, bacteria were resuspended in sterile water (*L. pneumophila*) or RPMI (*L. longbeachae*). The optical density (OD) at 600 nm was measured to estimate the concentration, and bacterial suspensions were diluted accordingly to perform *in vivo* infections.

### Cryptococcus neoformans

*C. neoformans* experiments were conducted using the strain H99. This clinical isolate was grown in minimal medium composed of dextrose (15 mM), MgSO_4_ (10 mM), KH_2_PO_4_ (29.4 mM), glycine (13 mM), and thiamine-HCl (3 M), as previously described (28). To ensure the virulence of the H99 strain, serial passages in C57BL/6 mice were performed before the fungal cells were used in experiments.

### Mice

C57BL/6 (JAX Stock No. 000664), *Casp1/11^—/—^* (29)*, Pycard^—/—^* (Asc KO) (30)*, Asc/Casp1/11^—/—^* (31), *Il1r1^—/—^* (JAX Stock No. 004754)*, Tlr2^—/—^* (JAX Stock No. 004650)*, Tlr4^—/—^* (32)*, Tlr9^—/—^* (33)*, Myd88^—/—^* (JAX Stock No. 009088)*, Ccr2^-/-^ (34), Tnfr1/2^—/—^* (35, 36)*, Il12p40^—/—^* (JAX Stock No. 002692)*, Ifng^—/—^* (JAX Stock No. 002287)*, Il17ra^—/—^* (37)*, Il-23p19^—/—^* (38)*, Ighm^—/—^* (B cell KO) (JAX Stock No. 002288), *Ciita^—/—^* (*CD4 KO)* (JAX Stock No. 003239), and *B2m^—/—^* (CD8 KO) (JAX Stock No. 002087) mice were bred and maintained at the Animal Facility of the University of São Paulo at Ribeirão Preto.

### Infections and experimental protocols

**Fig 1:**
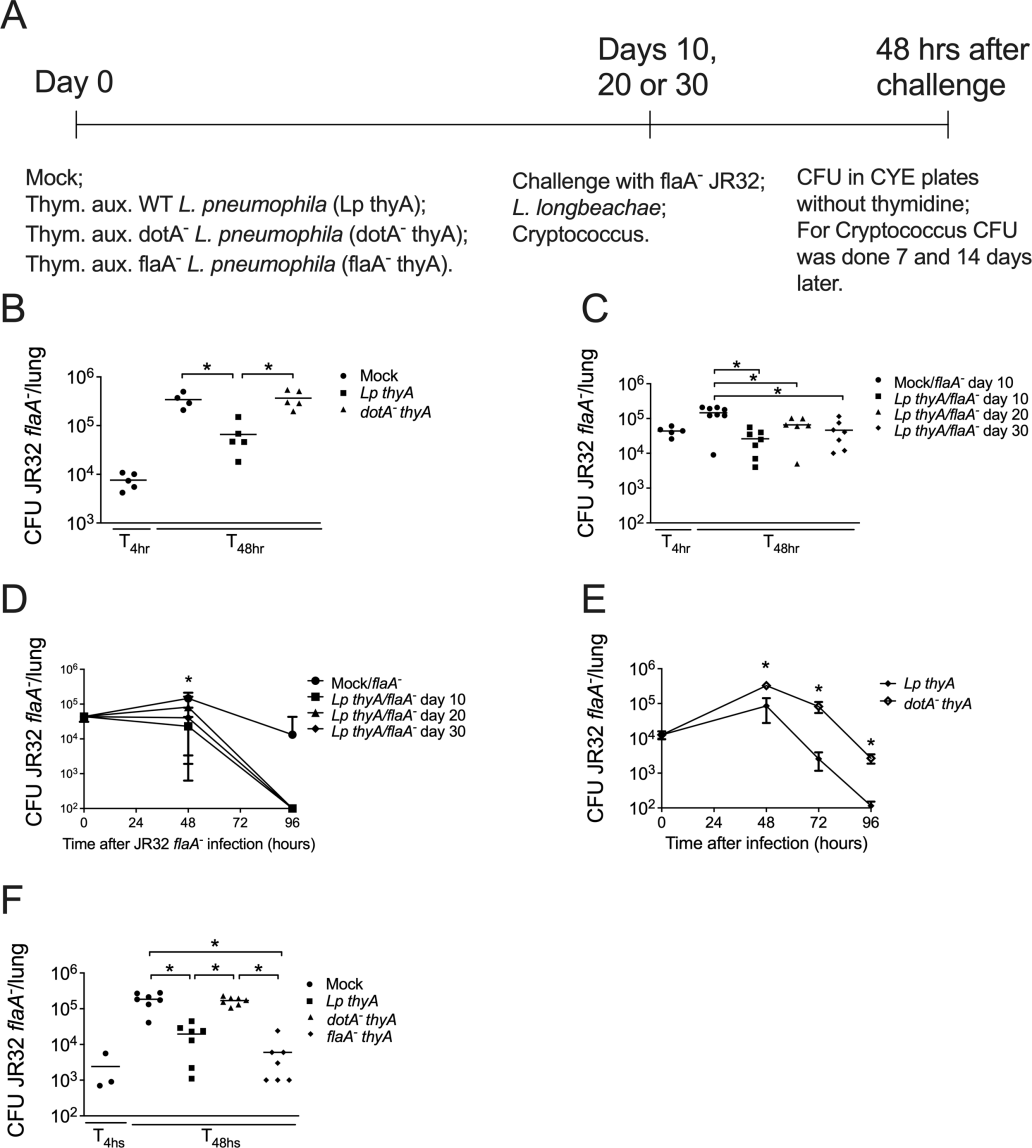
The Dot/Icm type IV secretion system is important for the induction of resistance against a second infection. (**A**) Summary of the general experimental approach used in this study. C57BL/6 mice were infected with thymidine auxotroph (Thym. aux.) *Legionella pneumophila* WT (*Lp thyA*), *dotA^-^* (*dotA^-^ thyA*) or *flaA^-^* (*flaA^-^ thyA*) at day 0. After 10, 20, or 30 days, mice were challenged with virulent JR32 *flaA^-^ L. pneumophila* that effectively replicates in C57BL/6 lungs, and CFU levels were determined after 48 h challenge. (**B**) The mice were Mock treated (PBS) or infected with *Lp thyA* or *dotA^-^ thyA* bacteria (Day 0), and after 10 days challenged with JR32 *flaA^-^* (Day 10). At 4 or 48 h after challenge, CFU in the mouse lungs was estimated. (**C**) Mice were Mock treated or infected with *Lp thyA* at day 0 and challenged with JR32 *flaA^-^* at days 10, 20, or 30. At 4 or 48 h after challenge, CFU in the mouse lungs was estimated. (**D**) Mice were Mock treated or infected with *Lp thyA* at day 0 and challenged with JR32 *flaA^-^* at days 10, 20, or 30. At 48 or 96 h after challenge, CFU in the mouse lungs was estimated. (**E**) Mice were infected with *Lp thyA* or *dotA^-^ thyA* bacteria at day 0 and challenged with JR32 *flaA^-^* at day 10. At 48, 72, or 96 h after challenge, CFU in the mouse lungs was estimated. (**F**) Mice were Mock treated or infected with *Lp thyA, dotA^-^ thyA,* or *flaA^-^ thyA* bacteria at day 0 and challenged with JR32 *flaA^-^* at day 10. At 4 or 48 h after challenge, CFU in the mouse lungs was estimated. Each dot represents a single animal, and the horizontal lines represent the averages. (**D and E**) Data show the average ± SD of 3–7 animals per group. (**B, C, and F**) *P < 0.05 compared with indicated groups, ANOVA followed by Bonferroni. (**D**) *, ^#^ and ^$^*P* < 0.05 compared with JR32 *flaA-* day 10, 20, and 30, respectively, ANOVA followed by Bonferroni. Data are one representative experiment of five (**B**) and two (**C–F**) performed with similar results.

For *in vivo* infections, female or male mice of 6–8 weeks were anesthetized with ketamine and xylazine (100 mg/kg ketamine; 10 mg/kg xylazine) by intraperitoneal administration, followed by intranasal inoculation of the corresponding bacteria diluted in phosphate-buffered saline (PBS). The animals were infected at day 0 and challenged at day 10, 20, or 30. Briefly, at day 0, mice were infected with thymidine auxotroph WT, *dotA^-^* or *flaA^-^* mutant *L. pneumophila* (1 × 10^5^/mouse), neither of which can replicate intracellularly (Fig. 1A). At day 10, 20, or 30, depending on the experiment, mice were challenged with JR32 *flaA^-^* strain (1 × 10^5^/mouse), *L. longbeachae* (1 × 10^5^/mouse for CFU and 1 × 10^7^/mouse for survival rate), or *C. neoformans* (1 × 10^5^/mouse for CFU and 1 × 10^4^/mouse for survival rate, both via intratracheal). Two days after infection with JR32 *flaA^-^* or *L. longbeachae,* mice were euthanized in a CO_2_ chamber, and the lungs were collected, homogenized in sterile water using a tissue homogenizer (Power Gen 125; Thermo Scientific, Waltham, MA, USA), diluted, and plated on CYE for determination of the number of colony-forming units (CFU) (Fig. 1A). *Cryptococcus neoformans* burden was quantified in lung homogenates, and measurements were evaluated 7 and 14 days post-infection. A sample of homogenate was diluted in sterile PBS buffer (pH 7.2), and aliquots of 100 µL were plated in Sabouraud agar medium. After 48 h of incubation at 30°C, the CFU was counted, and the concentration of yeast cells/mL was calculated. For the experiments on survival rate, the survival of mice was recorded every 12 h for 10 (*L. longbeachae*) or 20 days (*C. neoformans*). At the end of 10 or 20 days, surviving mice were euthanized.

### Flow cytometry

To determine the number of cells in the lungs after infection (Day 0) with thymidine auxotroph WT (*Lp thyA*), *dotA^-^ thyA* or mock control, mice were euthanized 2 and 10 days later, and the lungs were harvested. Lungs were digested for 1 hour at 37°C with Collagenase I (Life Technologies, Invitrogen, Basel, Switzerland), and a single-cell suspension was prepared by forcing the organ through a 70-μm strainer. Red blood cells were lysed using ACK lysis buffer. To block unspecific staining, cells were incubated for 30 minutes in a solution of rabbit serum diluted in PBS (5%). Subsequently, the cells were labeled with the following Abs: Alexa Fluor 700-labeled anti-mouse CD45, FITC-labeled anti-mouse CD11b (clone M1/70), PerCP-labeled anti-mouse Ly6G (clone 1A8), PE-labeled anti-mouse Ly6C (clone HK1.4), PECy7-labeled anti-mouse CD11c, APC-labeled anti-mouse F4/80 or PECy7-labeled CD3, PerCP-labeled anti-mouse CD4, APC-Cy7-labeled anti-mouse CD8, and FITC-labeled anti-mouse TCRγδ. Live/Dead Fixable Blue Cell Stain Kit (Life Technologies) was used to exclude dead cells. After staining, the cells were washed, fixed (1% paraformaldehyde in PBS, Sigma-Aldrich, St. Louis, MO), and the acquisition was carried out in a FACSCanto II cytometer (BD Biosciences). Data were analyzed by FCS Express V3 (*De Novo* Software).

### Statistical analyses

Statistical analyses were performed using Prism 5.0 software (GraphPad, San Diego, CA). Unpaired Student t test or one-way analysis of variance (ANOVA) followed by Bonferroni correction was used to compare the experimental groups. The survival rates were analyzed by the Mantel-Cox log-rank test. Differences were considered statistically significant at *P* < 0.05.

## RESULTS

### The type IV secretion system is essential for inducing host protective immunity

In the present study, we investigate whether infection with thymidine auxotroph *L. pneumophila* (*Lp thyA*), which cannot replicate intracellularly, would protect against secondary infection and the role of the Dot/Icm type IV secretion system (T4SS) in this process. For that purpose, mice were intranasally infected with *Lp thyA* or thymidine auxotroph *dotA^-^ L. pneumophila* (*dotA^-^ thyA*) (1 × 10^5^/mouse), and after 10 days, they were challenged with JR32 *flaA^-^* strain (1 × 10^5^/mouse) (Fig. 1A). We used *flaA^-^* mutants in the challenge experiments because, in the absence of flagellin, *Legionella* bypasses the NAIP5/NLRC4 inflammasome and effectively replicates in the lungs of C57BL/6 mice (16, 25). Interestingly, we found that infection with WT, but not *dotA^-^* mutants*,* significantly protected against the secondary infection with JR32 *flaA^-^,* as observed by reduced bacterial loads in the lung 48 hours after JR32 *flaA^-^* infection (Fig. 1B). Moreover, this effect was long-lasting, since mice challenged 20 or 30 days after the first infection were still able to control bacterial replication in the lung in comparison to mock-infected mice (Fig. 1C). In line with these findings, mice that were infected with *Lp thyA* and challenged 10, 20, or 30 days later with JR32 *flaA^-^* completely cleared lung bacteria at 96 hours after the secondary infection with JR32 *flaA^-^*. However, mock or *dotA^-^ thyA L. pneumophila-*infected mice still presented bacteria in the lung at this time point (Fig. 1D and E). Altogether, these results demonstrate that protection against virulent bacteria is induced by a first exposure with attenuated *L. pneumophila* and requires the expression of type IV secretion system.

Next, we investigated whether the protection conferred by Dot/Icm T4SS requires the secretion of flagellin into host cells, which is known to cause strong innate immune activation via the NAIP5/NLRC4 inflammasome. Interestingly, the first infection with flagellin-mutant bacteria (*flaA^-^ thyA*) still protected the mice against the second infection with JR32 *flaA^-^* (Fig. 1F), indicating that NAIP5/NLRC4 is not essential for this protection. Together, these results indicate that an intact type IV secretion system, rather than flagellin, induces protection against secondary *L. pneumophila* infection.

### Thymidine auxotroph *L. pneumophila* induces transient inflammation in the lung, resolved after 10 days of infection

**Fig 2:**
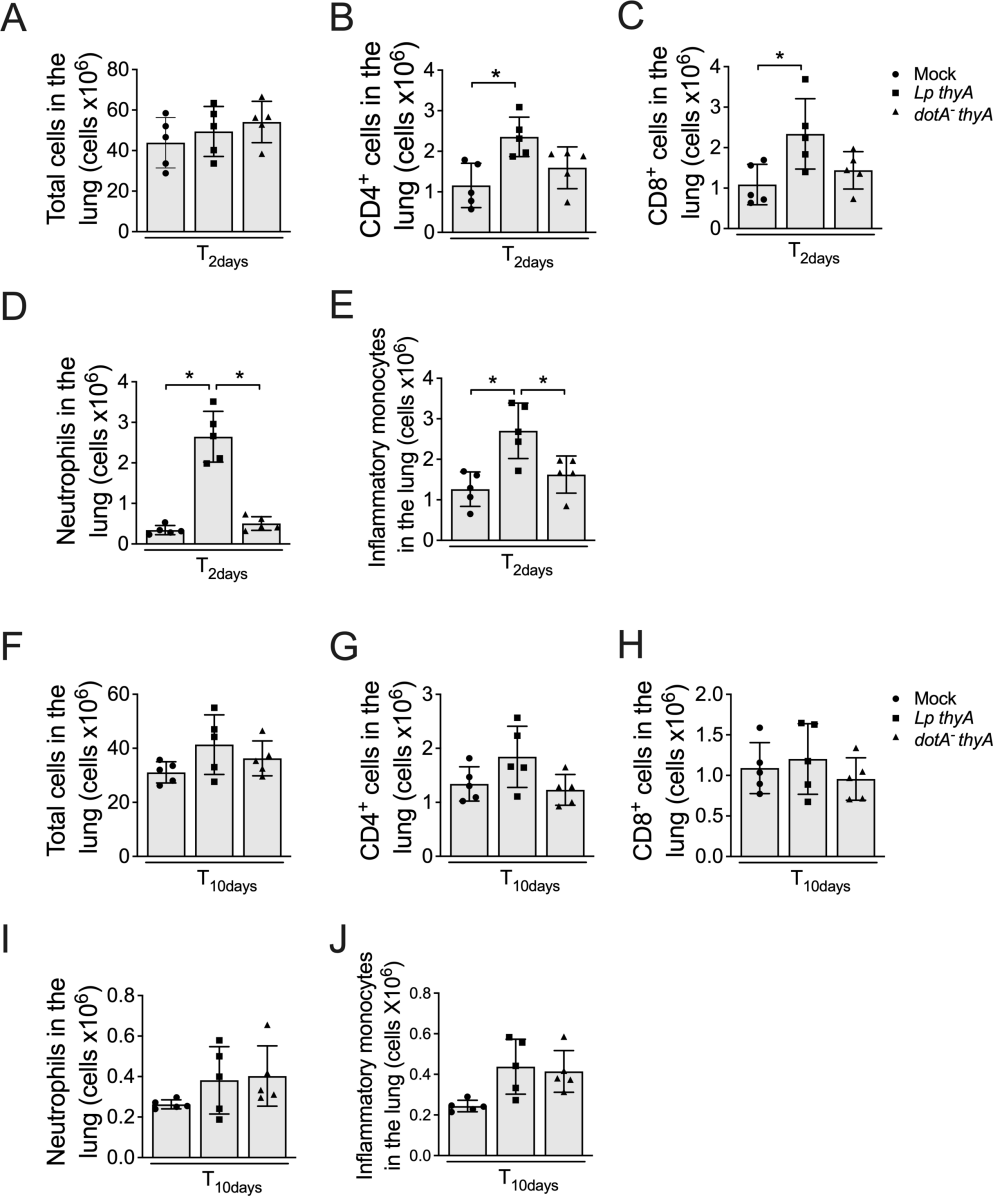
Infection with non-replicative *L. pneumophila* induces Dot/Icm-dependent inflammation in the lung after 2 days that resolves after 10 days. C57BL/6 mice were Mock treated (PBS) or infected with thymidine auxotroph *Legionella pneumophila* WT (*Lp thyA*), *dotA^-^* (*dotA^-^ thyA*). Mice were infected for 2 days (**A–E**) or 10 days (**F–J**), and the lungs were collected and stained for FACs analyses phenotyping. The graphs show the total number of cells (**A and F**), the numbers of CD4^+^ T cells (CD3^+^/CD4^+^; **B and G**), CD8^+^ T cells (CD3^+^/CD8^+^; **C and H**), neutrophils (CD11b^+^/Ly6G^+^; **D and I**), and inflammatory monocytes (Ly6G^-^/CD11b^+^/CD11c^-^/Ly6C^+^; **E and J**). Data show the average ± SD of 5 animals per group. *P < 0.05 as determined by ANOVA followed by Bonferroni. Data are one representative experiment out of two experiments performed with similar results.

The finding that *Lp thyA,* but not *dotA^-^ thyA L. pneumophila,* triggers protection against a secondary infection prompted us to investigate the inflammatory status in the lung of mice after the first infection with non-replicating thymidine auxotroph *L. pneumophila*. Thus, we infected mice with *Lp thyA* and *dotA^-^ thyA* and evaluated the proportion of selected inflammatory cells after 2 and 10 days. We found no differences in the total number of cells between mock-, *Lp thyA,* and *dotA^-^ thyA-*infected mice at both 2 and 10 days post-infection (Fig. 2A and F). However, after 2 days of infection, we found Dot/Icm-dependent increase numbers of CD4+ (Fig. 2B) and CD8+ (Fig. 2C), neutrophils (Fig. 2D), and inflammatory monocytes (Fig. 2E) in mice lungs. These data indicate that Dot/Icm activities promoted the stimulation of immune cells in the lungs after 2 days of infection, and this process occurs regardless of bacterial replication. However, different than shown after 2 days of infection, we found that at 10 days after infection, the numbers of CD4 and CD8 T cells, neutrophils, and inflammatory monocytes were similar in mice infected with *Lp thyA* or *dotA^-^ thyA L. pneumophila* bacteria (Fig. 2G through J). These data indicate that although the T4SS triggers increased acute inflammation in the lungs after 2 days of infection, this response reaches similar levels at day 10, when we performed the challenges (Fig. 1). These data indicate that at 10 days after infection, the inflammatory status of the lungs is similar regardless of the infection of *Lp thyA* or *dotA^-^ thyA L. pneumophila* at day 0, suggesting that the T4SS-induced protection is likely the result of induction of adaptive immunity rather than the result of residual recruitment of inflammatory cells.

### MyD88, CCR2, and CD4, but not inflammasomes, are required for a type IV secretion system-induced immune response

**Fig 3:**
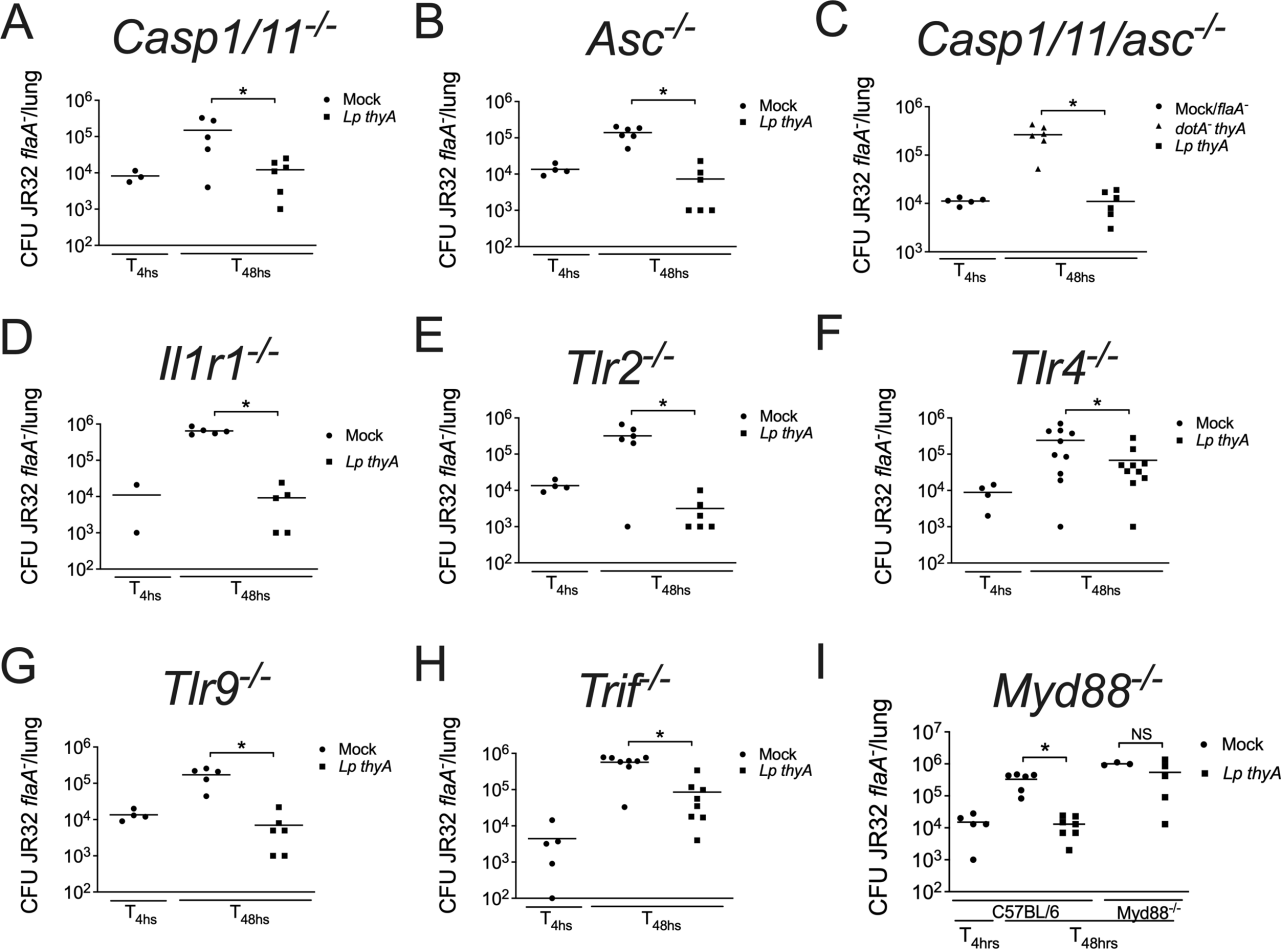
MyD88 is required for Dot/Icm-mediated acquired immunity. Mice were Mock treated (PBS) or infected with *Lp thyA* or *dotA^-^ thyA* bacteria for 10 days and challenged with JR32 *flaA^-^*. At 4 or 48 h after challenge, and CFU in the mouse lungs was estimated in infections performed in (**A**) *Casp1^—/—^*, (**B**) *Asc^—/—^*, (**C**) *Casp1/11^—/—^/Asc^—/—^*, (**D**) *Il1r1^—/—^*, (**E**) *Tlr2^—/—^*, (**F**) *Tlr4^—/—^*, (**G**) *Tlr9^—/—^*, (**H**) *Trif^—/—^*, and (**I**) C57BL/6 and *Myd88^—/—^* mice. Each dot represents a single animal, and the horizontal lines represent the averages. *P < 0.05 comparing the indicated groups, unpaired Student t test. Data are one representative experiment of one (**A–H**) and four (**I**) performed with similar results.

To understand the role of the innate immune system in the induction of host resistance against subsequent infection, we next infected mice deficient in selected innate immune receptors. Several studies have demonstrated that the activation of inflammasomes effectively contributes to the restriction of *L. pneumophila* multiplication (reviewed in reference 18). Thus, we initially infected mice deficient in Caspase-1/11 (Fig. 3A), ASC (Fig. 3B), Casp1/11/ASC (Fig. 3C), and IL-1R (Fig. 3D) and observed that the first exposure to *Lp thyA L. pneumophila* was still able to induce protection against the subsequent infection in these animals, when compared to mice that were first infected with *dotA^-^ thyA* bacteria or mock. We further investigated the role of specific *Toll*-like receptors that are known to be activated by *L. pneumophila*. We found that TLR2 (Fig. 3E), TLR4 (Fig. 3F), TLR9 (Fig. 3G), and the TLR3/4 adaptor molecule TRIF (Fig. 3H) are dispensable for inducing host resistance against the subsequent infection, as observed by reduced bacterial loads in the lung of mice that were first infected with *Lp thyA L. pneumophila* compared to mock. By contrast, the absence of Myd88 signaling abrogated the protection mediated by first exposure to *Lp thyA* bacteria (Fig. 3I). It is possible that multiple innate immune sensors in addition to IL1R that signal via MyD88 operate in this process, and deletion of individual receptors results in compensation via the other receptors. Nonetheless, our data with *Myd88^—/—^* mice indicate that this pathway is key for the Dot/Icm-mediated protection.

**Fig 4:**
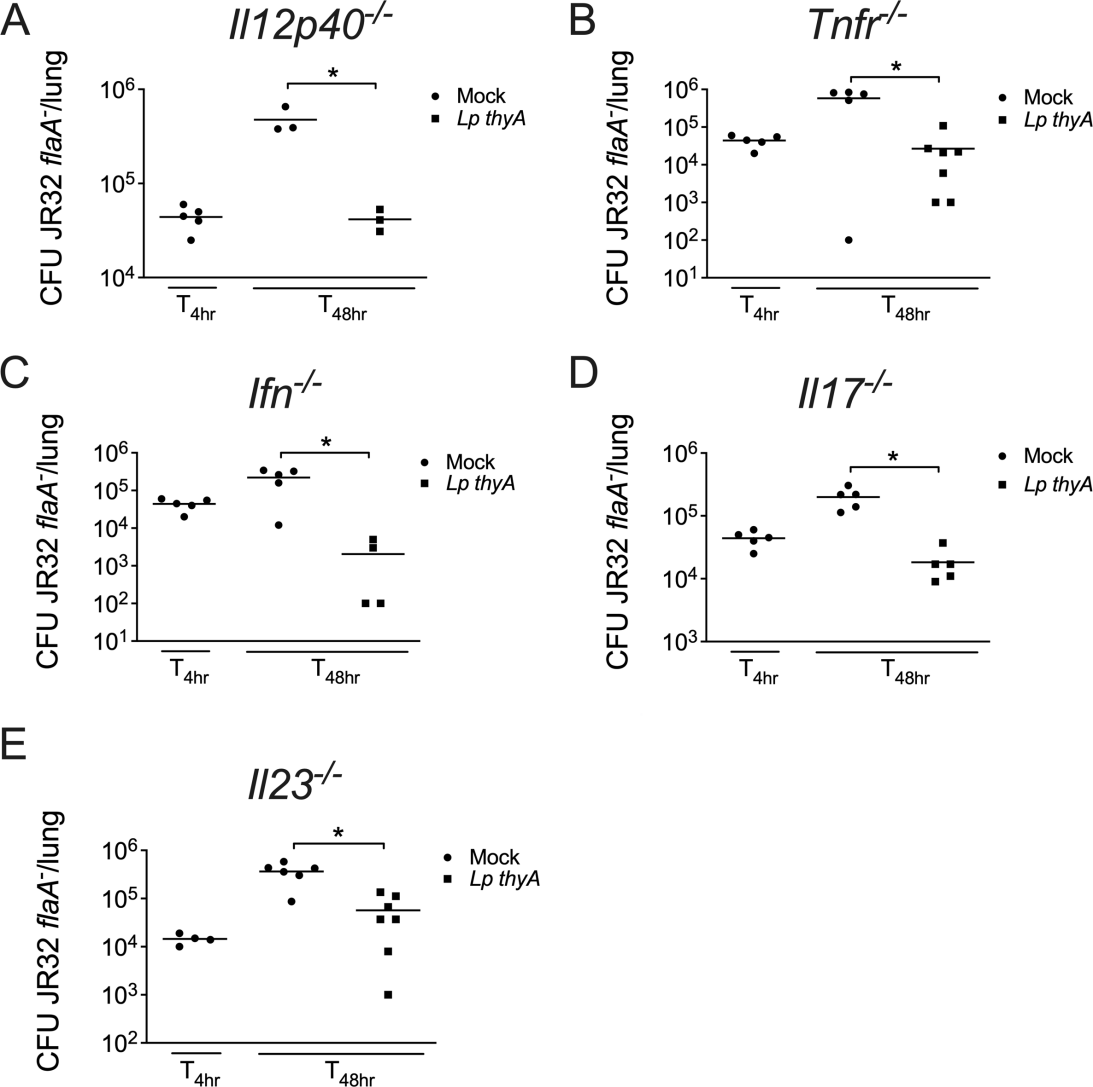
IL-12, TNF-α, IFN-γ, IL-17, and IL-23 are dispensable for Dot/Icm-mediated acquired immunity. Mice were Mock treated (PBS) or infected with *Lp thyA* or *dotA^-^ thyA* bacteria for 10 days and challenged with JR32 *flaA^-^*. At 4 or 48 h after challenge, CFU in the mouse lungs was estimated in infections performed in (**A**) *Il12p40^—/—^*, (**B**) *Tnfr^—/—^*, (**C**) *Ifna^—/—^*, (**D**) *Il17^—/—^*, and (**E**) *Il23^—/—^* mice and the CFUs were measured. Each dot represents a single animal, and the horizontal lines represent the averages. *P < 0.05 comparing the indicated groups, unpaired Student t test. Data are one representative experiment of one (**A and D**) and two (**B, C, and E**) performed with similar results.

Given that IL-12 and TNF-α are important cytokines produced upon MyD88 signaling and critical for immunity, we then investigated whether these cytokines are required for the protection induced by an initial *Legionella* infection. By analyzing the bacterial loads in mice deficient in IL-12p40 (Fig. 4A) and TNF receptor (Fig. 4B), we still observed protection against secondary infection with JR32 *flaA^-^*, indicating that these cytokines are not essential in host resistance. We also investigated the involvement of cytokines not directly produced upon MyD88 signaling in this process. Likewise, we found no involvement of IFN-γ, IL-17RA, or IL-23 in host protection upon infection (Fig. 4C through E).

**Fig 5:**
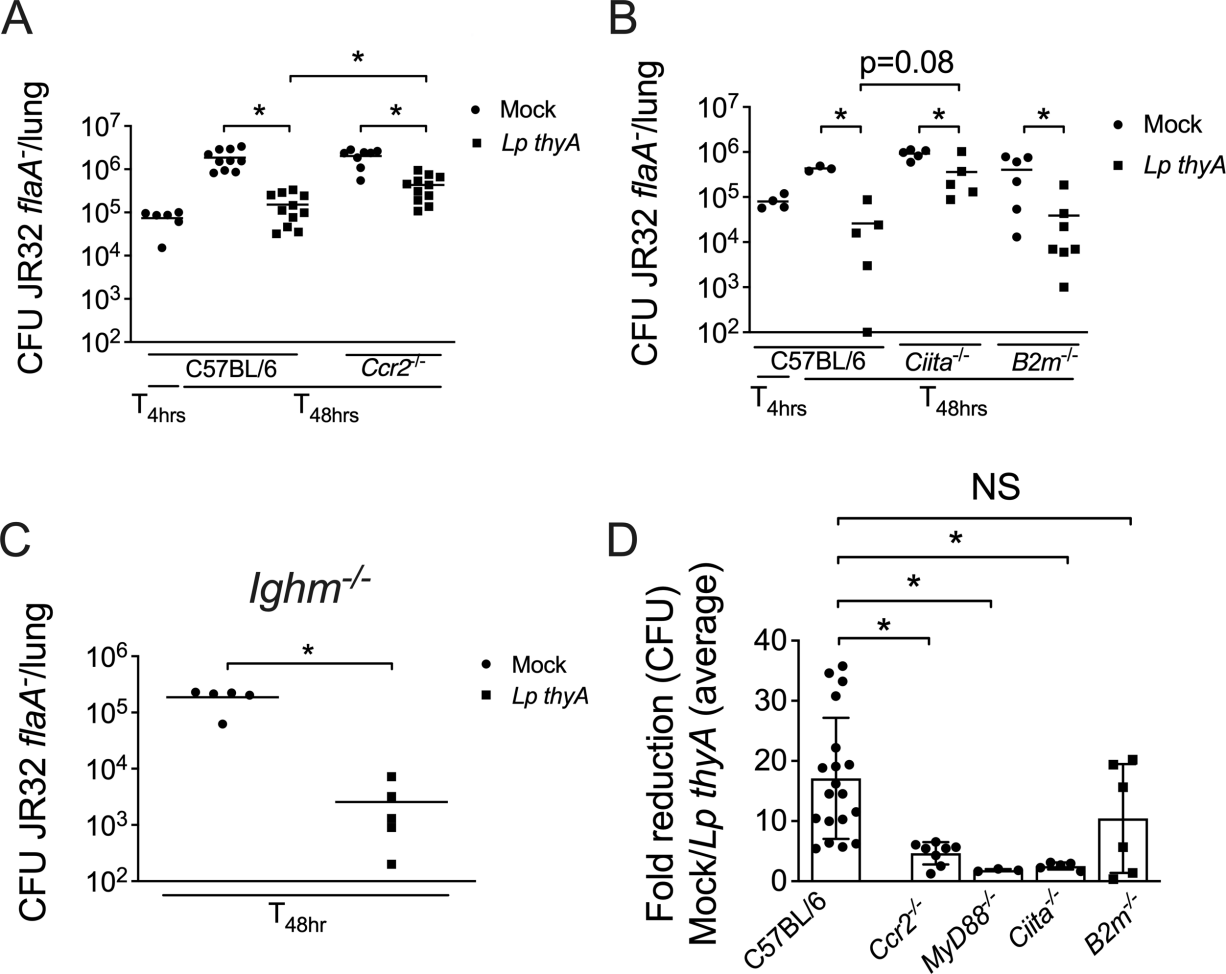
Dot/Icm-mediated protection requires acquired immunity. C57BL/6, *Ccr2^—/—^* (**A**)*, Ciita^—/—^* (CD4 KO)*, B2m^—/—^* (CD8 KO) (**B**), and *Ighm^—/—^* (B KO) (**C**) mice were Mock treated (PBS) or infected with *Lp thyA* or *dotA^-^ thyA* bacteria for 10 days and challenged with JR32 *flaA^-^*. At 4 or 48 h after challenge, CFU in the mouse lungs was estimated. Each dot represents a single animal, and the horizontal lines represent the averages. Fold reduction in CFU was calculated by dividing the CFU value of each mock-infected mouse by the average obtained for *L. pneumophila thyA*-infected group (**D**). Data are presented as average ± SD, and each dot represents an individual animal. *P*  <  0.05 for comparisons between the indicated groups, as determined by unpaired Student t test. The data shown are from one representative experiment of two (**A**), three (**B**), and one (**C**) independent experiments with similar results. Panel D displays fold reduction in CFU from the same experiments shown in panels A–C.

Since chemokines induced by MyD88 signaling, as well as chemokine receptors such as CCR2, have previously been shown to play important roles in the host response to *L. pneumophila* (8, 39–42), we investigated whether CCR2^+^ monocytes are required for Dot/Icm-mediated protection against secondary infection. We found that protection in *Ccr2^—/—^* mice was less effective than in C57BL/6 mice, indicating that both MyD88 signaling and CCR2^+^ cells contribute to Dot/Icm-mediated protection against a secondary *L. pneumophila* infection (Fig. 5A).

We further evaluated the requirement of components of adaptive immunity, such as MHCII and MHCI, as well as the involvement of T and B cells. Interestingly, although MHCI/CD8 deficient mice *(B2m)* were still protected against a subsequent infection, the difference between *Lp thyA* and Mock-infected *MhcII^—/—^* (CD 4 KO*, Ciita*) mice was half log smaller in relation to the difference observed in C57BL/6 mice (Fig. 5B). By contrast, the single deficiency on B cells does not abrogate protection against a subsequent infection (Fig. 5C). To further confirm the involvement of CD4^+^ T cells, we analyzed the fold change in CFU between C57BL/6 mock controls and the *L. pneumophila* thyA-infected group and compared these results to those from the *Ciita^—/—^* group. Notably, the reduction in CFU was less pronounced in the *Ciita^—/—^* group compared to C57BL/6 mice, indicating a role for CD4^+^ T cells in Dot/Icm-mediated host protection. By contrast, there was no significant difference in fold change between C57BL/6 and *B2m^—/—^* mice, suggesting that CD8^+^ T cells are dispensable for this protection. To further illustrate the roles of MyD88, CCR2, and CD4 in host protection, we expressed the data shown in Fig. 5A through C as fold reduction in CFU. We observed statistically significant differences between C57BL/6 and *Myd88^—/—^*, *Ccr2^—/—^*, and *Ciita^—/—^* mice, but not when compared to *B2m^—/—^* mice (Fig. 5D). Altogether, our results indicate that both innate and adaptive immune systems are involved in T4SS-mediated host protection against secondary infection with *L. pneumophila*.

### Type IV secretion system is involved in generating specific and nonspecific protective immunity

**Fig 6:**
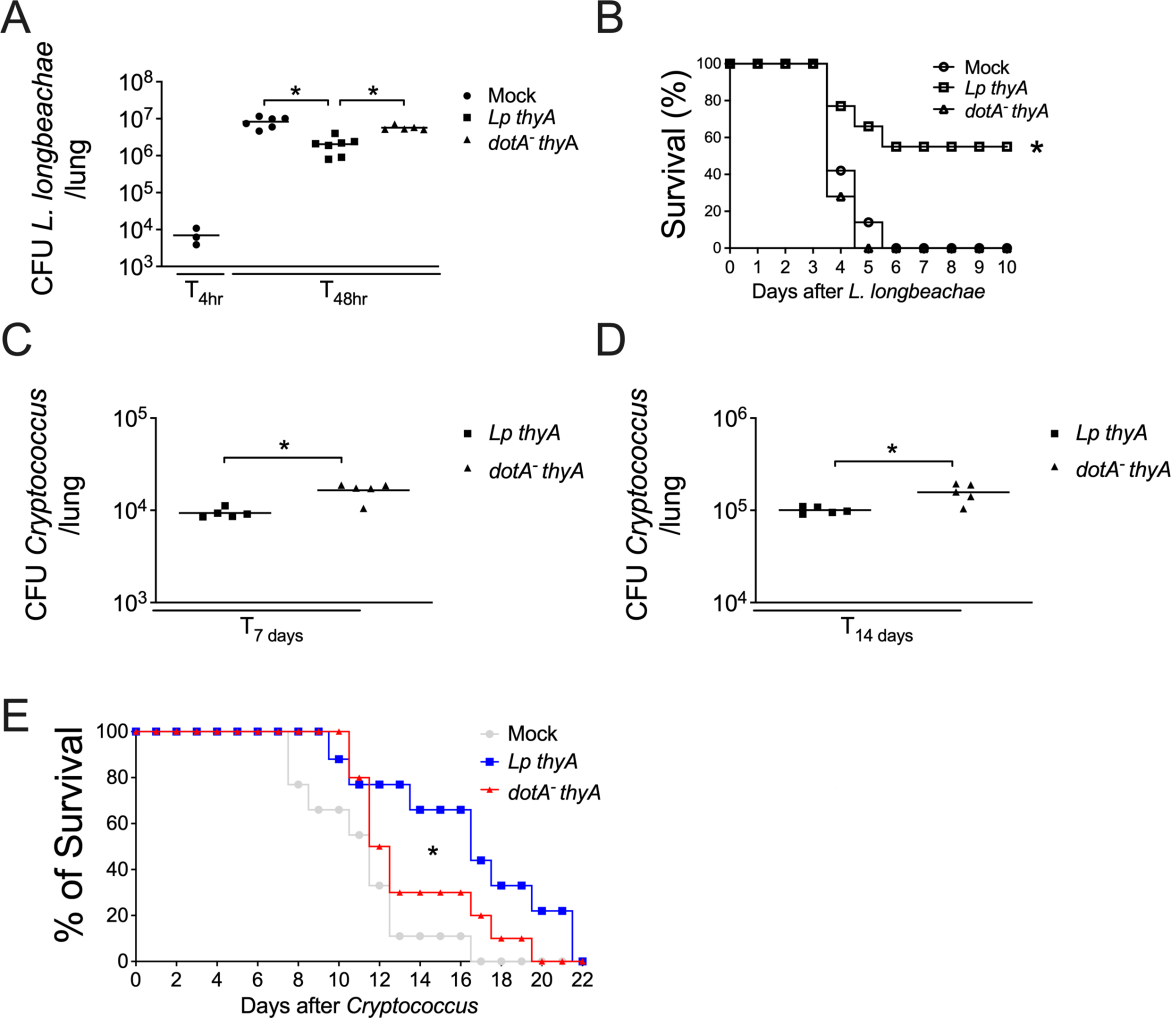
Type IV secretion system is involved in the generation of specific and nonspecific protective immunity. C57BL/6 mice were Mock treated (PBS) or infected with *Lp thyA* or *dotA^-^ thyA* bacteria for 10 days and challenged with *Legionella longbeachae* or *Cryptococcus neoformans*. (**A**) CFU in the lung at 4 and 48 hours post-challenge with *L. longbeachae*. (**B**) Percentage survival of mice challenged with *L. longbeachae.* (**C and D**) *C. neoformans* CFU in the lung at 7 (**C**) and 14 (**D**) days post-challenge. (**E**) Percentage survival of mice challenged with *C. neoformans*. In CFU experiments, each dot represents a single animal, and the horizontal lines represent the averages. *P < 0.05 comparing the indicated groups, ANOVA followed by Bonferroni. For each survival experiment, 7–10 animals per group were used and analyzed by the Mantel-Cox log-rank test. Data are one representative experiment of two performed with similar results.

Our previous data suggest that both innate and adaptive immunity are involved in the protection mediated by the Dot/Icm system. To further assess this process, we investigated whether the Dot/Icm-mediated protection is pathogen-specific. To test this hypothesis, we performed the first infection with *Lp thyA*, *dotA^-^ thyA L. pneumophila* or mock and performed the subsequent infection with *Legionella longbeachae,* a capsulated bacterium that is lethal to mouse (43–45). Interestingly, mice that were previously infected with *Lp thyA L. pneumophila* were also protected against a subsequent infection with *Legionella longbeachae*, as observed by reduced bacterial loads in the lungs 48 hours after *L. longbeachae* inoculation (Fig. 6A) and by the increased survival of mice first exposed to *Lp thyA L. pneumophila* (Fig. 6B). As *L. pneumophila* and *L. longbeachae* share antigen similarities, we next used a non-bacterial pathogen to evaluate the specificity of this immune response. Thus, we first infected with *Lp thyA* or *dotA^-^ thyA L. pneumophila* and performed the subsequent infection using the pathogenic fungus *Cryptococcus neoformans,* which is well known to be lethal to mice (46). We found that mice that were first infected with *Lp thyA L. pneumophila* were protected against secondary infection with *C. neoformans* as observed by decreased fungal loads in the lungs after 7 and 14 days of *C. neoformans* infection (Fig. 6C and D). The initial infection with *Lp thyA L. pneumophila* also delayed the *C. neoformans*-induced death in comparison with mice that were first infected with *dotA^-^ thyA L. pneumophila* (Fig. 6E). Thus, T4SS-mediated immune activation can protect the mice against secondary infections by a fungal pathogen, consistent with the induction of trained immunity. Collectively, these data indicate that *Legionella* T4SS triggers both the generation of a specific protective immunity and nonspecific immunity, consistent with trained immunity.

## DISCUSSION

In the present study, we demonstrated that a bacterial type IV secretion system is essential for the generation of a protective response against a secondary infection, with the participation of components of both innate and adaptive systems. These T4SS-mediated protective responses against reinfection are long-lasting and pathogen-specific, but they can also protect the host against a non-related pathogen, consistent with the concept of trained immunity. Until the last decade, the prevailing view was that acquired immunity was only mediated by adaptive immunity. Upon the encounter of a pathogen, the adaptive immunity builds an immunological response to that specific microorganism, and a subsequent encounter with the same pathogen leads to clonal expansion of memory T and/or B cells, triggering a rapid and effective response (23). However, the greater protection against reinfection in plants and invertebrates, organisms that do not have an adaptive immune system, and the fact that some infections and vaccines can induce protection against other pathogens challenged this dogma (47). In 2011, Netea and colleagues proposed the term trained immunity to describe the capacity of the innate immunity to respond in a rapid and stronger manner to a secondary nonspecific stimulus (48). More recently, it was proposed that this broad protection of the innate immune system can be acquired via a crosstalk between adaptive and innate memory responses (49). Here, we demonstrated that the protection conferred against secondary infection with pathogenic bacteria or fungi after a first infection with an attenuated strain of *L. pneumophila* is also dependent on both innate and adaptive immunity. Specifically, this protective response is dependent on MyD88 and partially dependent on CCR2 and is not specific to the microorganism used during the initial infection. This suggests a mechanism resembling trained immunity. Consistently, several studies have utilized *Ccr2^—/—^* mice to demonstrate the involvement of innate immune cells in trained immune responses (50–52). On the other hand, the response observed in our study is also dependent on the adaptive immunity, as *Ciita^—/—^* mice, lacking CD4^+^ T cells, are less protected against secondary infection. However, previous studies have shown that adaptive immune cells, such as T cells, can be required to initiate trained immunity (49, 53). Therefore, the partial dependence on CD4^+^ T cells for Dot/Icm-mediated host protection could reflect a role for adaptive immunity or T-cell-mediated induction of trained immunity. Importantly, our data indicate that the induction of protective immunity relies on the expression of *Legionella pneumophila* Dot/Icm, a feature that may be useful for therapeutic approaches based on trained immunity.

The Dot/Icm type IV secretion system used by *L. pneumophila* and other bacteria is of fundamental importance for bacterial pathogenicity through the secretion of several effector proteins directly into the cytoplasm of host cells (reviewed in reference 4). It has been shown that this system is required for the induction of Th17 response against *L. pneumophila* (20). Accordingly, we are also showing that the Dot/Icm type IV secretion system is involved in the generation of a protective long-term immunity against a subsequent bacterial infection. However, we did not search for specific Dot/Icm effector molecules nor putative structural components of the bacteria responsible for the phenotype observed in this study.

MyD88 is the canonical adaptor for inflammatory signaling pathways downstream of members of the Toll-like receptor (TLR) and interleukin-1 (IL-1) receptor (IL-1R) families. MyD88 signaling can lead to the production of pro-inflammatory or anti-inflammatory cytokines and chemokines. However, although we demonstrated that MyD88 is involved in the protection against a secondary infection, when we analyzed the individual participation of IL-1R, TLR2, 4, and 9 and the cytokines TNF and IL-12, we did not observe impairment of adaptive immunity in the absence of these molecules individually. These data suggest the existence of a redundancy in signaling pathways of innate immunity for the induction of acquired immunity in response to bacterial virulence determinants. Altogether, our study demonstrates the importance of the interplay between innate and adaptive immunity in specific recognition of bacterial virulence factors, such as the T4SS. Our data contribute to our understanding of overall immunity against pathogenic intracellular bacteria and also indicate that the inclusion of virulence determinants may be critical for the successful development of vaccines against bacterial pathogens.
